# Investigation of anti-oxidative stress *in vitro* and water apparent diffusion coefficient in MRI on rat after spinal cord injury *in vivo* with *Tithonia diversifolia* ethanolic extracts treatment

**DOI:** 10.1186/1472-6882-14-447

**Published:** 2014-11-18

**Authors:** Chi-Long Juang, Fei Shish Yang, Ming Shuang Hsieh, Hu-Yi Tseng, Su-Chiu Chen, Hsiao-Chuan Wen

**Affiliations:** Department of Medical Imaging and Radiological Technology, Yuanpei University, Hsinchu, 30015 Taiwan; Department of Radiological Technology, Mackay Memorial Hospital, Taipei, Taiwan; Department of Radiological Technology, Taipei City Hospital, Yangming Branch, Taipei, Taiwan; Department of Optometry, Yuanpei University, Hsinchu, 30015 Taiwan

**Keywords:** Spinal cord injury, Apparent diffusion coefficient, *Tithonia diversifolia* ethanolic extracts, DPPH, LDH

## Abstract

**Background:**

Spinal cord injury (SCI)-induced secondary oxidative stress associates with a clinical complication and high mortality. Treatments to improve the neurological outcome of secondary injury are considered as important issues. The objective of the current study is to evaluate the anti-oxidative effect of *Tithonia diversifolia* ethanolic extracts (TDE) on cells and apply the pharmacological effect to SCI model using a MRI imaging algorism.

**Methods:**

The anti-oxidation properties were tested in a 2,2-diphenyl-1-picrylhydrazyl (DPPH) radical scavenging assay. Rat liver cells (clone-9) were treated with various doses of TDE (0 ~ 50 μg/ml) before exposed to 250 μM H_2_O_2_ and cell survival was determined by MTT and LDH assays. We performed water apparent diffusion coefficient (ADC) map in MR techniques to investigate the efficacy of TDE treatment on SCI animal model. We performed T5 laminectomy and compression (50 g, 1 min) to induce SCI. PHILIP 3.0 T MRI was used to image 24 male Sprague–Dawley rats weighing 280–320 g. Rats were randomly divided into three groups: sham group, SCI group, SCI treated with TDE group. The MRI images were taken and ADC were acquired before and after of treatment of TDE (50 mg/kg B. W. orally, 5 days) in SCI model.

**Results:**

TDE protected clone-9 cells against H_2_O_2_-induced toxicity through DPPH scavenging mechanism. In addition, SCI induced the increase in ADC after 6 hours. TDE treatment slightly decreased the ADC level after 1-week SCI compared with control animals.

**Conclusion:**

Our studies have proved that the cytoprotection effect of TDE, at least in part, is through scavenging ROS to eliminate intracellular oxidative stress and highlight a potential therapeutic consideration of TDE in alternative and complementary medicine.

## Background

*Tithonia diversifolia* (*T. diversifolia*), a bushy perennial weed, is commonly found in Nigerian fields, wastelands and roadsides in Taiwan. The plant is primarily used for ornamental purposes, and further studies have demonstrated its effects on treatment of diabetes mellitus, diarrhea, fever, hematomas, hepatitis, hepatomas, malaria and wounds [1, 2]. The *T. diversifolia* contains bioactive compounds which have been proved to possess anti-inflammatory effects [3]. In addition, phytochemical investigations of *T. diversifolia* have identified the existence other bioactive compounds, such as cadinane, chromene, eudesmane, flavone, germacrane and rearranged eudesmane derivatives [4–11]. The extracts from aerial and stem of *T. diversifolia* have demonstrated anti-inflammatory and liver protective effects in rats against paw edema induced by carrageenan and acute hepatic damage induced by CCl_4_, respectively [2]. The extracts from *T. diversifolia* and the major effective compound sesquiterpenoid, the tagitinin C, have been identified as a potential anti-cancer therapeutic reagent both in vitro and in vivo [12–16]. Other than its anti-cancer activity, the sesquiterpenoid that extracted from other species shows its anti-inflammatory effect via inhibition of oxidative stress both *in vitro* and *in vivo* in the treatment of psoriasis [17]. However, whether the anti-inflammatory effect from *T. diversifolia* extracts is mediated by its ability of anti-oxidative stress remains to be elucidated.

Increasing numbers of small animal models are in use in the field of neurological research. To evaluate the functions of soft tissues, Magnetic Resonance Imaging (MRI) can provide an excellent method for non-invasive imaging of nervous tissue. Specialized small-bore animal MRI scanners are available for high-resolution MRI of small rodents’ tissue, but the major drawback of this equipment is its high cost. Several research groups have performed clinical MR scanners for imaging small animal models. To achieve a reasonable spatial resolution at an acceptable signal-to-noise ratio with these scanners, several requirements regarding to sequence parameters need to be matched considerately. Thus, the goal of this study is seeking to successfully apply MRI for examining small rodents’ spinal cord injury using a standard clinical 3 T scanner and to keep instrumental cost low. Our improvement including the use of apparent diffusion coefficient (ADC), which measures the diffusion of water molecules within central nervous system, is applied in this study. A low value of ADC indicates that the nerve fiber tracts are well-organized, while a high value of ADC indicates that these tracts are damaged and disorganized. Therefore, while evaluating an acute ischemic stroke, ADC images may play a crucial role in identifying the degree of damage due to its higher water content in injured area.

The damage that occurs in the central nervous system (CNS) following trauma is due to secondary effects of glutamate excitotoxicity, Ca2^+^ overload, and oxidative stress [18, 19]. Oxidative stress activates mechanisms that result in a neutrophil-mediated inflammation which can also cause secondary damage. Mechanisms of oxidative stress are involved in lipid peroxidation and production of reactive oxidative species production. We suggest that decreasing oxidative stress greatly reduces the amount of secondary damage due to trauma. Aggressive nutritional support following CNS trauma can also contribute to maximizing antioxidant defenses. Furthermore, we suggest that natural supplements such as herbs have the potential to be therapeutically effective because of their free radical quenching, iron chelating, and anti-inflammatory properties.

Decrease in oxidative phosphorylation processes in CNS may induce neurological deficits. Natural antioxidants may help the antioxidant capacity of the organism to prevent from disease associated with reactive oxygen species (ROS) and other oxidative damage inducing agents. In this study, we sought to explore anti-oxidative stress *in vitro* with *T. diversifolia* Ethanolic Extracts (TDE) and the pharmacological effect of TDE on ADC in MRI on rat after SCI *in vivo*.

## Methods

### Plant extraction

The leaves of *T. diversifolia* were collected in Hsin-Chu, Taiwan, in January 2011. A voucher specimen was deposited in the Department of Medical Imaging and Radiological Technology, Yuanpei, University, Hsin-Chu, Taiwan. Collected plant materials were dried and ground into powder using a grinder and screened through a 20-mesh sieve (aperture of 0.94 mm). The powder of *T. diversifolia* leaves (200 g) was extracted 3 times with 1 L ethanol at room temperature and sonicated for the duration of 5 h (100 min for each time). The plant residue was filtered through a 10 μm cartridge paper, and the ethanolic extracts were combined and concentrated by a rotary vacuum evaporator. The detailed extraction of *T. diversifolia* was followed by Liao *et al*. [13, 14], modified by Tseng *et al*. [20] and reported in previously paper [13–15, 20]. Ming-Shueh Shieh, a Professor in Department of Environmental Engineering and Health, who major in identity of plant, preceded the identity of the plant material used in our study prior to its use. The ethanolic extracts of the leaves of *T. diversifolia* were named TDE.

### Cell lines and cultures

Clone-9 (normal rat liver cell) were cultured in Dulbecco modified Eagle medium (DMEM) (Gibco, NY, USA) supplemented with 10% fetal bovine serum (FBS), 2 mM glutamine, 100 U/mL penicillin,100 mg/mL streptomycin sulfate and 1 mM sodium pyruvate then incubated at 37°C at an atmosphere of 5% CO_2_/O_2._.

### Scavenging ability on DPPH radical

The 2,2-diphenyl-1- picrylhydrazyl (DPPH) test was used to evaluate the free radical scavenging capacity (RSC) of TDE in cell free systems according to a published procedure [21] using ascorbic acid as positive control substance. Different concentrations of TDE were tested and absorbance was measured at 490 nm after 30 min of reaction with a stock solution of DPPH.

### Cell viability assay

Cell viability was determined by 3-(4, 5-dimethylthiozol-2-yl)-2, 5-diphenyltetrazolium bromide (MTT) spectrophotometric analysis. Cells (2 × 10^4^ cells/mL) were seeded in 24-well culture plates and allowed to adhere 24 hr. After 24 hr of incubation, the extracts were added, and the plates were incubated for another 24 hr. Cells were washed once before adding 50 mL of FBS-free medium containing MTT (0.5 mg/mL). After 3 hr of incubation at 37°C, the medium was discarded, and the formazan blue in the cells was dissolved in DMSO. The optical density (O. D.) was measured at 540 nm. The experiments were repeated three times.

### Determination of protection of H_2_O_2_-induced damage in normal liver cells by the MTT and LDH Assays

The protective effect of TDE against toxicity induced by H_2_O_2_ in normal rat liver cells (clone 9) was carried out using MTT and lactate dehydrogenase (LDH) assays. Cells were seeded as described above and treated with TDE (10, 30, 50 μg/ml) for 24 hr. Clone 9 cells after treatment were then exposed to phosphate buffered saline (PBS) free medium containing 250 μM H_2_O_2_ for 1 hr. The MTT assay and LDH assay was performed after hydrogen peroxide exposure. The assay of LDH release was determined by a commercial kit from Roche^®^ following the manufacturer’s instruction.

### Compression SCI model

All animal experimental manipulations were under taken in accordance with the National Institutes of Health Guide for the Care and Use of Laboratory Animals, with the approval of the Experimental Animal Ethical Committee of Taipei Mackay Memorial Hospital. All animals were anesthetized with an overdose of sodium pentobarbital and sacrificed immediately after finishing experiment. Sprague-Dawley rats weighing between 280 and 320 g were anesthetized by intramuscular (inraperitoneal) administration of Zoletil 50 and Ropum (2:1). A laminectomy was performed at vertebral level T5, leaving the dura undisturbed. The T4 and T6 transverse processes were clamped in a spinal compression frame and the compression was applied by a modified aneurysm clip which produced a compression force of 50 grams and lasted for 1 min. After being induced spinal cord injury or sham control, all rats were placed in warmed cages to recover from anaesthesia. The rats were given free access to food and tap water, and were maintained on a 12 hr light/dark cycle at 21 ± 1°C.

### Animal MRI

The rats were examined in a 3 T whole-body MR system (Achieva X-series; Philips, Eindhoven, Netherland). A wrist coil (SENSE-wrist-8) was used to obtain all MR images, allowing parallel imaging. To avoid movement, rats were placed into a plastic holder. Animals imaged with T1-, T2-, and diffusion-weighted sequences. ADC maps were constructed from the diffusion-weighted data. A fast T2- weighted spin echo sequence was used as localizer. A transverse T1-weighted spin-echo [3000 msec/100 msec (repetition time/echo time), field of view (FOV) of 81.3 mm^2^] and a T2-weighted turbo spin-echo sequence TR 2000 msec/TE40 msec were performed with a section area of 1 mm^2^. A DW-MRI echo-planar sequence was performed with a *b*-values = 800 sec/mm^2^. The following parameters were used for this sequence: TR = 2870 milliseconds, TE = 111 milliseconds and a total time of acquisition of 2:35 minutes. Gradient strength was adjusted for *b*-value. ADC maps were calculated from the native diffusion images with the built-in software tools of the MRI scanner. The measured fractional anisotropy (FA) directional diffusivities derived from diffusion weighted image (DWI) and the diffusion tensor imaging (DTI) was applied in capture details of the anatomy for tractography.

### Statistical analysis

All data are expressed as mean ± SEM of three independent experiments in *in vitro* study. Every independent experiment included three wells of cells. Data were processed by one-way analysis of variance (ANOVA) tests using SPSS. Intergroup comparisons were made using paired *t*-test or students’ *t* -test. Differences were considered significant when *p* < 0.05.

## Results

**Figure 1:**
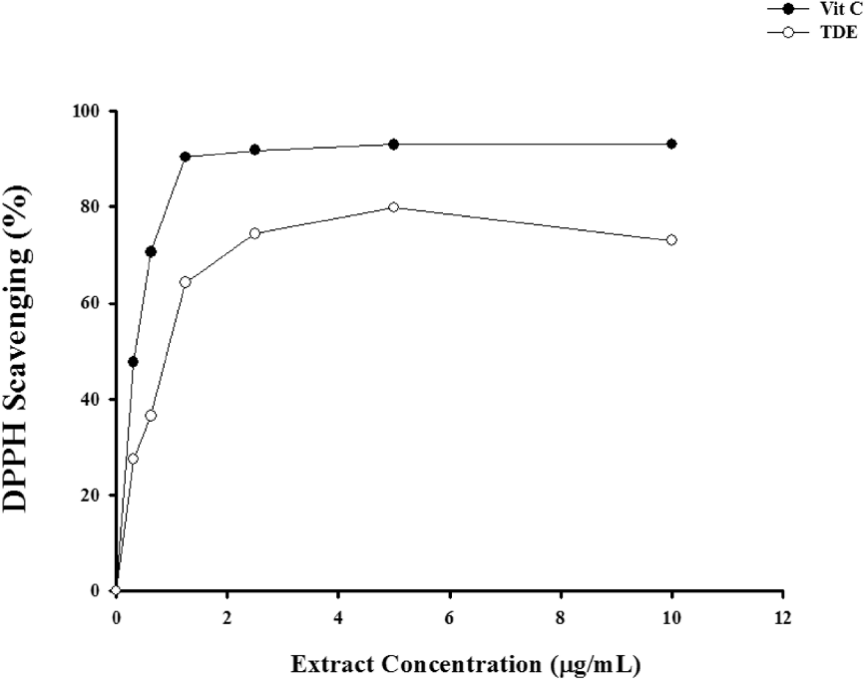
**Free radical scavenging profile of TDE expressed as % of DPPH scavenging compared to ascorbic acid.** IC_50_ values for TDE and ascorbic acid were 0.93 ± 0.2 and 0.48 ± 0.1 μg/ml respectively.

We first examined the antioxidative effect of TDE by using an *in vitro* cell free system. We performed DPPH to test the free RSC of TDE. The use of vitamin C was as a positive control. Free radical scavenging profile of TDE was expressed as % of DPPH inhibition compared to ascorbic acid. The result showed that the IC50 values for TDE and ascorbic acid were 0.93 ± 0.20 and 0.48 ± 0.10 μg/ml, respectively (see Figure 1), suggesting a free radical scavenging ability of TDE.

**Figure 2:**
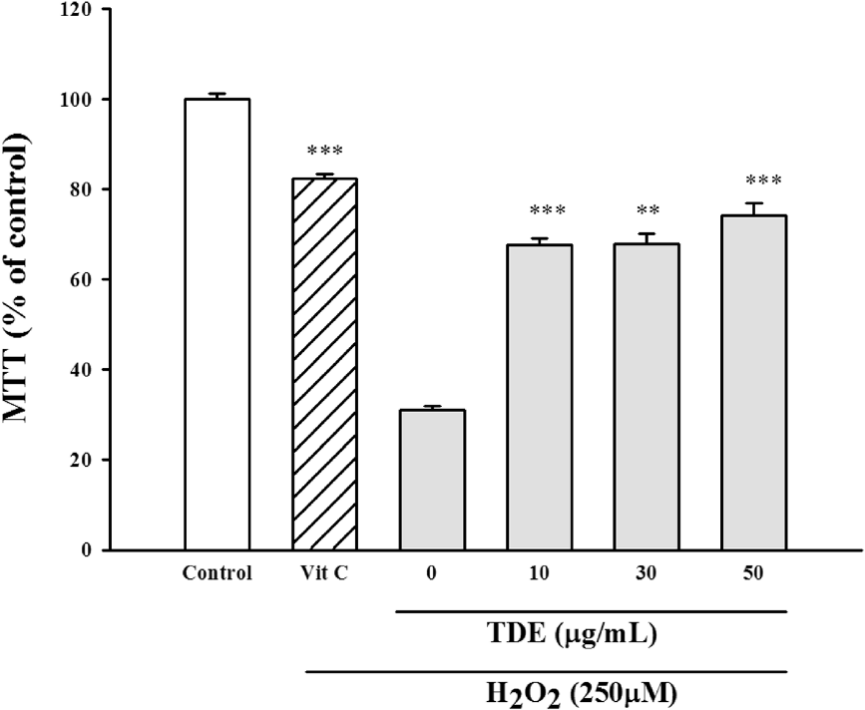
**Cytoprotective effect of TDE on hydrogen peroxide induced toxicity in clone 9 cells by MTT assays.** **p < 0.01; ***p < 0.001 versus hydrogen peroxide-treated cells. Control group refers to non-treated cells.

**Figure 3:**
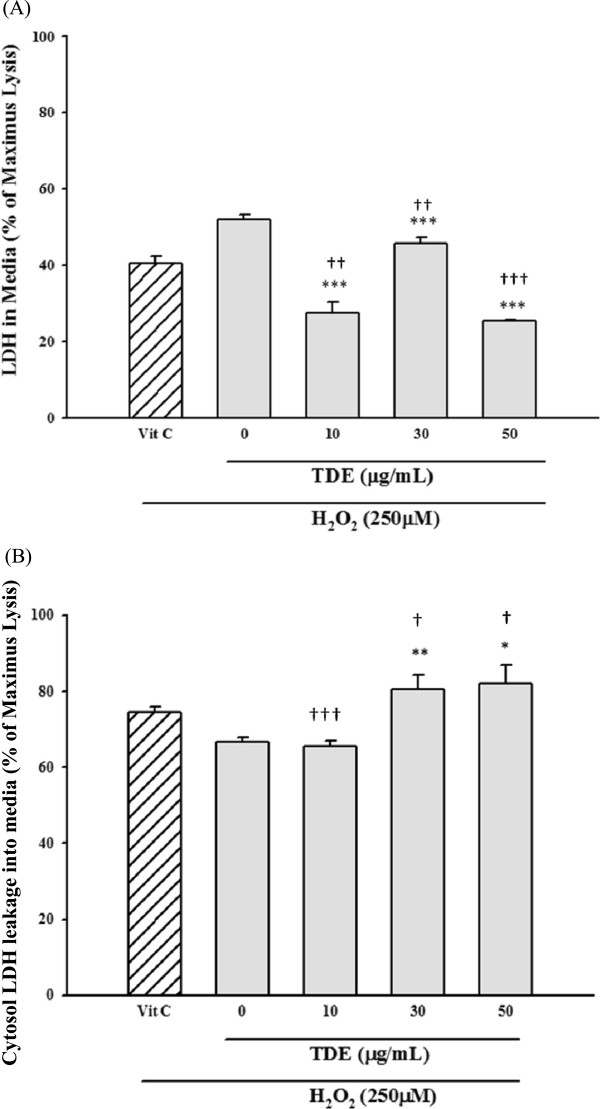
**Cytoprotective effect of TDE on hydrogen peroxide induced toxicity in clone 9 cells by LDH assays.** LDH released in medium was assayed in **(A)** and LDH leakaged from cytosol was assayed in **(B)** after triton X-100 treatment. **p < 0.01; ***p < 0.001 versus hydrogen peroxide-treated cells. + p < 0.01 versus vit C group. Control group refers to cell free medium.

We then tested the anti-oxidative effects of TDE in cell culture system. The clone 9 was pretreated with TDE of different doses (0-50 μg/ml), followed by exposing the cells in 250 μM H_2_O_2_. Cell survival was detected by MTT assay and LDH release assay. Vitamin C treatment was used as a positive control (Figure 2). We found that treatment of H_2_O_2_ alone resulted in significant decrease of cell viability. Pretreatment of TDE protected H_2_O_2_-induced decrease of cell viability (Figure 2). Similar results were found in LDH release assay, where H_2_O_2_-induced release of LDH into medium from cytosol was significantly reduced by TDE protection (Figure 3A) and cytosolic LDH was significantly higher with TDE treatment than without TDE treatment (Figure 3B). These results suggest that TDE possesses anti-oxidative effect against H_2_O_2_-induced cytotoxicity.

**Figure 4:**
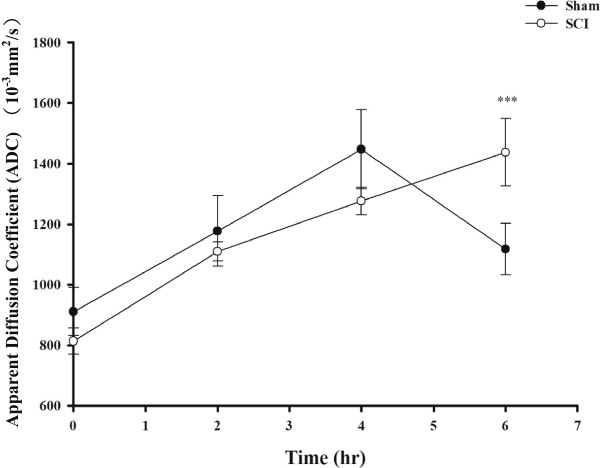
**Time course of spinal cord injury (SCI) on apparent diffusion coefficient (ADC).** Rats were induced SCI by T5 laminectomy and compression (50 g, 1 min). ADC map was used to assessment the injured level. Data are presented as mean ± S.E.M of six rats. Significance for the time-dependent effect after SCI treatment was calculated by ANOVA. *P < 0.05, significantly different from untreated control samples.

**Figure 5:**
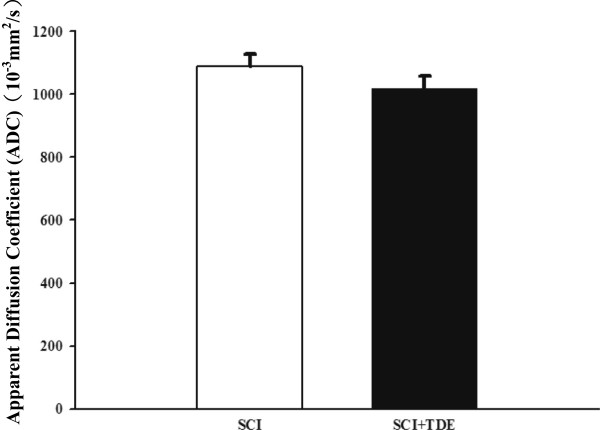
**Effect of TDE Treament after SCI.** TDE treatment slightly decreased the ADC level in 1 week after SCI compared with no TDE treatment in animal study.

Given that TDE has demonstrated a potential ant-oxidative and free radical scavenging effect in the *in vitro* system, we are seeking whether this plant extract can be performed to an *in vivo* system. One of the known *in vivo* systems with oxidative stress is spinal cord injury [18, 19]. To assist the data evaluation, we also performed ADC to analyze MRI images and examined the SCI grading. At different time points (0, 2, 4, 6 hr) after SCI, rats were anesthetized and analyze the ADC value using MRI. The results showed SCI resulted in significant increase in ADC after 6 hr (Figure 4), suggesting an increase of cell permeability after SCI. To further confirm the SCI injury model was successfully executed, we then performed DTI to evaluate the damage level of fiber tracts after SCI [20]. TDE treatment slightly decreased the ADC level in 1 week after SCI compared with no TDE treatment in animal study (Figure 5).

## Discussion

Development of early detectors of treatment response can provide information which would be of significant benefit for both experimental and clinical trials. These issues underscore the need for using noninvasive imaging to facilitate the evaluation of the responsiveness of preclinical therapeutic studies. In this regard, MRI techniques are earlier and more sensitive and predictive methods to determine treatment efficacy during the initial course of treatment would be extremely valuable for facilitating therapeutic protocol planning. Serial study of such MR parameters as DWI, ADC, and T2-weighted imaging may provide information on the pathophysiological mechanisms of acute ischemic stroke [19, 22–25]. Our studies showed ADC was elevated in the acute stage after SCI 6 hr because of increased extracellular water permeability *via* decrease of Na-K pump activity, influx of Ca^2+^, water and efflux of K^+^. The information provides us that the time to improve the neurological outcome of SCI is very important.

Compression models of rat SCI mainly use either a modified aneurysm clip or a static compression platform, and the aneurysm clip has been described in detail [26–28]. A static compression platform has been used in several studies [16, 29] but has not been characterized so fully [30]. In this paper we have therefore used a T5 static compression model of SCI using diffusion tensor imaging (DTI) to evaluate the damage level of the fiber tracts and this data has successfully published [20]. Huang *et al*. found that static compression SCI results in a prolonged profile of cell loss [31]. In contrast, the temporal profile of cell loss in the rat contusion SCI model is very different. Both neuronal and oligodendrocyte loss after contusion injury are acute events. The differences between the compression and contusion models are important to study the pathophysiology of SCI. The delayed cell death in our compression models provides a good opportunity to assess the efficacy of neuroprotective interventions.

**Figure 6:**
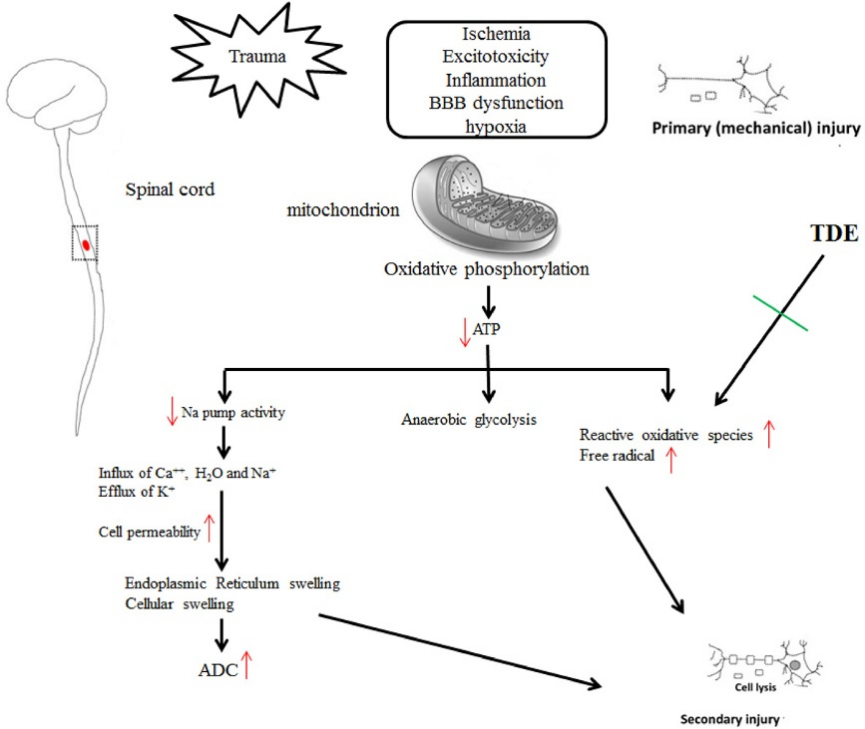
**The schematic results of current study.** We proposed that TDE protects cells against hydrogen peroxide or radical scavenging-induced toxicity, and that an antioxidant mechanism through ROS scavenging may be in part responsible for cells neuroprotection.

*T. diversifolia* has been shown to possess anti-inflammatory and analgesic activity [2]; however, the neuroprotective or antioxidant effect has never been established. In the present study, we have evaluated the free radical scavenging effect, anti-oxidative stress of TDE in the *in vitro* model system and the results are summarized in Figure 6. Furthermore, ooxidative stress in SCI is known to induce neuronal damage and results in serial dysfunctions. In current study, our study has also suggested a potential therapeutic effect of TDE in SCI.

Consequently, the consumption of plants with neuroprotecive effects in the complementary therapy may represent a strategy to prevent from a wide range of disease. Given the composition of TDE is complex, there has been marked variability in animal study. That is the reason that the data showed only 4 of 6 in SCI decrement assayed by ADC after TDE treatment. It has been reported that contain sesquiterpenoids and flavonoids [12] which may have neuroprotective effect. In our phytochemical screening, TDE reveals flavonoids (350.46 rutin equivalent μg/mg), and phenols (1273.01 galic acid equivalent μg/mg). Isolated constituents from TDE have previously been demonstrated to exert their effect in neuronal cells [13–15]. For example, studies regarding to *T. diversifolia* reveal anti-inflammatory effect [2]. In this context, TDE may act through ROS scavenging in part in response to reduce intracellular oxidative stress to exert its neuroprotective effect.

Spinal cord injury such as ischemia, excitotoxicity, blood brain barrier dysfunction and hypoxia cause primary or mechanical injury. Previous reports about CNS trauma can induce oxidative stress and produce free radical and then cause secondary injury which induces paraspinal edema, stroke and other neurological damages. Trauma-induced edema formation in SCI is related with clinical complication and high mortality. In this animal study, we used Diffusion Tensor Imaging (DTI) to confirm the consistency effect of damage level of fiber tracts after SCI [20] and ADC map in MR techniques for studying the efficacy of TDE treatment.

## Conclusion

These findings demonstrate the protective effect of TDE against oxidative stress may act through free radical scavenging properties. TDE may be considerate as an interest in complementary medicine in the future. Further research is needed to define health benefits and clinical effects of TDE.
